# Moderate Exercise in Spontaneously Hypertensive Rats Is Unable to Activate the Expression of Genes Linked to Mitochondrial Dynamics and Biogenesis in Cardiomyocytes

**DOI:** 10.3389/fendo.2020.00546

**Published:** 2020-08-19

**Authors:** Clara Quiroga, Georthan Mancilla, Ingrid Oyarzun, Anita Tapia, Mia Caballero, Luigi A. Gabrielli, Denisse Valladares-Ide, Andrea del Campo, Pablo F. Castro, Hugo E. Verdejo

**Affiliations:** ^1^Laboratorio de Señalización Cardiovascular, División de Enfermedades Cardiovasculares, Facultad de Medicina, Pontificia Universidad Católica de Chile, Santiago, Chile; ^2^Advanced Center for Chronic Diseases (ACCDIS), Santiago, Chile; ^3^Instituto de Ciencias de la Salud, Universidad de O'Higgins, Rancagua, Chile; ^4^Laboratorio de Fisiología y Bioenergética Celular, Departamento de Farmacia, Facultad de Química y de Farmacia, Pontificia Universidad Católica de Chile, Santiago, Chile

**Keywords:** exercise, mitochondrial dynamics, hypertension, cardiac remodeling, heart

## Abstract

Hypertension (HTN) is a public health concern and a major preventable cause of cardiovascular disease (CVD). When uncontrolled, HTN may lead to adverse cardiac remodeling, left ventricular hypertrophy, and ultimately, heart failure. Regular aerobic exercise training exhibits blood pressure protective effects, improves myocardial function, and may reverse pathologic cardiac hypertrophy. These beneficial effects depend at least partially on improved mitochondrial function, decreased oxidative stress, endothelial dysfunction, and apoptotic cell death, which supports the general recommendation of moderate exercise in CVD patients. However, most of these mechanisms have been described on healthy individuals; the effect of moderate exercise on HTN subjects at a cellular level remain largely unknown. We hypothesized that hypertension in adult spontaneously hypertensive rats (SHRs) reduces the mitochondrial response to moderate exercise in the myocardium.

**Methods:** Eight-month-old SHRs and their normotensive control—Wistar-Kyoto rats (WKYR)—were randomly assigned to moderate exercise on a treadmill five times per week with a running speed set at 10 m/min and 15° inclination. The duration of each session was 45 min with a relative intensity of 70–85% of the maximum O_2_ consumption for a total of 8 weeks. A control group of untrained animals was maintained in their cages with short sessions of 10 min at 10 m/min two times per week to maintain them accustomed to the treadmill. After completing the exercise protocol, we assessed maximum exercise capacity and echocardiographic parameters. Animals were euthanized, and heart and muscle tissue were harvested for protein determinations and gene expression analysis. Measurements were compared using a nonparametric ANOVA (Kruskal-Wallis), with *post-hoc* Dunn's test.

**Results:** At baseline, SHR presented myocardial remodeling evidenced by left ventricular hypertrophy (interventricular septum 2.08 ± 0.07 vs. 1.62 ± 0.08 mm, *p* < 0.001), enlarged left atria (0.62 ± 0.1 mm vs. 0.52 ± 0.1, *p* = 0.04), and impaired diastolic function (E/A ratio 2.43 ± 0.1 vs. 1.56 ± 0.2) when compared to WKYR. Moderate exercise did not induce changes in ventricular remodeling but improved diastolic filling pattern (E/A ratio 2.43 ± 0.1 in untrained SHR vs. 1.89 ± 0.16 trained SHR, *p* < 0.01). Histological analysis revealed increased myocyte transversal section area, increased Myh7 (myosin heavy chain 7) expression, and collagen fiber accumulation in SHR-control hearts. While the exercise protocol did not modify cardiac size, there was a significant reduction of cardiomyocyte size in the SHR-exercise group. Conversely, titin expression increased only WYK-exercise animals but remained unchanged in the SHR-exercise group. Mitochondrial response to exercise also diverged between SHR and WYKR: while moderate exercise showed an apparent increase in mRNA levels of *Ppargc1*α*, Opa1, Mfn2, Mff*, and *Drp1* in WYKR, mitochondrial dynamics proteins remained unchanged in response to exercise in SHR. This finding was further confirmed by decreased levels of MFN2 and OPA1 in SHR at baseline and increased OPA1 processing in response to exercise in heart. In summary, aerobic exercise improves diastolic parameters in SHR but fails to activate the cardiomyocyte mitochondrial adaptive response observed in healthy individuals. This finding may explain the discrepancies on the effect of exercise in clinical settings and evidence of the need to further refine our understanding of the molecular response to physical activity in HTN subjects.

## Introduction

Exercise is a key element of optimal cardiovascular health: physical activity and exercise training improves cardiovascular fitness and reduces the risk of cardiovascular disease (CVD). The importance of exercise has been highlighted in most clinical practice guidelines, emphasizing the association between sedentary behaviors and cardiovascular morbidity and mortality (1). A key mediator of the beneficial effects of exercise training is the activation of mitochondrial adaptation mechanisms that allow skeletal muscles to sustain contraction and resist fatigue due to an improved ability to sustain aerobic ATP synthesis (2). However, mitochondrial adaptation to exercise is not restricted to skeletal muscle, but a systemic response that affects the entire organism (3).

Hypertension is the most common preventable CVD risk factor (4). Several clinical trials have probed the antihypertensive effect of exercise: in hypertensive adults, aerobic exercise training lowers blood pressure 5–7 mmHg, rivaling the effect of the first-line antihypertensives (5). Based on these results, exercise is recommended as a lifestyle modification for hypertension prevention and treatment; however, the type of exercise, as well as the optimal frequency, intensity, and duration is based on expert opinion and not on substantial evidence. This lack of agreement reflects a concerning fact: the robustness of epidemiologic evidence regarding the beneficial effect of exercise contrasts with our poor understanding of the mechanisms underlying its effects in specific conditions such as hypertension (6).

The Spontaneously Hypertensive Rat (SHR) model derives from selectively inbreeding Wistar–Kyoto rats (WKYR) with high blood pressures (5). The SHR is a well-characterized model of hypertension and provides the ideal setting for understanding the effects of lifestyle modifications such as exercise in a polygenic background, closely resembling human disease.

In our paper, we aimed to characterize the effect of moderate exercise in a well-known murine model of hypertension—the spontaneously hypertensive rat (SHR), under the hypothesis that hypertension reduces the mitochondrial response in the myocardium.

## Methods

### Reagents

Antibodies (anti-phospho-mTOR, mTOR, phospho-AKT, AKT, phospho-AMPK, AMPK) were acquired from Cell Signaling Technology (Danvers, MA, USA). Anti-OPA1, Protein Phosphatase Inhibitor Cocktail IV, and Protein Protease Inhibitor Cocktail (EDTA-free) were purchased from Abcam (Cambridge, MA, USA). T-PER™ Tissue Protein Extraction Reagent, TRIzol™ Reagent, and M-MLV reverse transcriptase were obtained from Thermo Fisher Scientific (Waltham, MA, USA). Anti-GAPDH antibody was purchased from Sigma-Aldrich Co. (St. Louis, MO, USA). Bradford's solution and PVDF membranes were from Bio-Rad Laboratories (Hercules, CA, USA). Secondary antibodies were obtained from Calbiochem (Burlington, ON, Canada). Organic and inorganic compounds, acids, and solvents were acquired from Merck (Darmstadt, Germany). Westar Supernova substrate was obtained from Cyanagen (Bologna, Italy). SensiFAST SYBR Hi-ROX was purchased from Bioline Meridian Biosciences (London, UK).

### Experimental Model

Male Spontaneously Hypertensive rats (SHR) and normotensive Wistar-Kyoto rats (WKYR) were purchased from Charles River Laboratories International, Inc. (Wilmington, MA, USA). Rats were kept in a temperature-controlled environment, on a 12-h light/dark cycle and *ad libitum* supply of food and water. All experiments and procedures were approved and monitored by Institutional Safety and Ethics Committees, Pontificia Universidad Católica de Chile, Chile.

Systolic and diastolic blood pressure (SBP and DBP, respectively) were assessed each week through a CODA^®^ Monitor non-invasive Blood Pressure System (Kent Scientific Corporation, Torrington, CT, USA) and recorded before, during and after starting the physical exercise protocol. Before starting the physical exercise protocol, 8-month-old rats were adapted to treadmill exercise with daily short sessions of 10 min at running speed set of 10 m/min by 14 days. Then, the animals were randomly assigned to four groups: untrained WKYR group (*n* = 8), trained WKYR group (*n* = 8), untrained SHR group (*n* = 10), and trained SHR group (*n* = 10). Both groups of untrained animals (WKYR and SHR) performed short treadmill sessions of 10 min at 10 m/min two times per week to keep them accustomed to the treadmill. The trained groups underwent moderate exercise on a treadmill five times per week with a running speed set at 10 m/min and 15° inclination. Each session lasted 45 min to complete 8 weeks. As described and standardized by Armstrong et al. we expected that exercise protocol reached a relative intensity of 70–85% of the maximum O_2_ consumption (7). One week after exercise protocol finishing, rats were euthanized, and heart and muscle tissue were harvested for protein determinations and gene expression analysis.

### Echocardiographic Study

After completing the exercise protocol, all the animals underwent an echocardiographic evaluation using a GE Vivid-I ultrasound scanner (GE Healthcare, Chicago, IL, USA) equipped with an i12L-RS transducer (4–10 MHz). Rats were anesthetized in a chamber with isoflurane and kept under isoflurane anesthesia (2%) in a 1:1 air-oxygen mixture. A two-dimensional parasternal long-axis view and short-axis view at the level of papillary muscles of the left ventricle (LV) were acquired using a high frame rate software (200–300 Hz). M-mode tracings were obtained from short-axis views of the LV at or just below the tip of the mitral valve leaflets, and at the level of the aortic valve and left atrium. A trained observer measured all LV structures using the leading-edge according to guidelines of the American Society of Echocardiography. Measurements represent the mean of at least five cardiac cycles on M-mode tracings. The following structural variables were measured: left atrium (LA) diameter, LV diastolic and systolic dimensions (LVDD and LVS.D, respectively), LV diastolic posterior wall thickness (PWT), LV diastolic septal wall thickness (SWT), and aortic diameter (AO) LV function was assessed by LV shortening fraction and by ejection fraction using the Teichholz' formula. Diastolic function was assessed by transmitral E and A wave using pulsed Doppler in four-chambers apical view (sweep speed of 100 mm/s) and by LA diameter.

### Histological Study

LV samples were fixed in formalin 10% and transferred to 1× PBS, followed by paraffin embedding. Hematoxylin & Eosin (H&E) staining was performed for cardiomyocyte size analyses and Masson's trichrome staining for fibrosis degree determination. Samples were observed with an Axio Imager Microscope (Carl Zeiss, Oberkochen, Germany) using a 40X objective. Images were recorded with Axio Vision software (v.4.8; Carl Zeiss) and analyzed with ImageJ software (National Institutes of Health, Bethesda, MD, USA). Paraffin-embedded tissue blocks, tissue cuts, and staining were performed in the Laboratory of Pathological Anatomy, Red de Salud UC-Christus, Santiago, Chile.

### Western Blot Analysis

Heart tissue was lysed with T-PER™ Reagent supplemented with phosphatase and protease inhibitor cocktails. Total protein concentration was determined by Bradford assay. Equal amounts of protein were loaded on 10% SDS polyacrylamide gels and then electro-transferred to PVDF membranes. After blocking, primary Abs, anti-phospho-mTOR Ser2448 (#5536), anti-mTOR (#2983), anti-phospho-AKT Ser473 (#4060), anti-AKT (#9272), anti-p-AMPK Thr172 (#2535), anti-AMPK (#2532), anti-OPA1 (#ab42364), and anti-GAPDH (#G9545), were incubated overnight at 4°C, and the appropriate horseradish peroxidase-conjugated secondary Abs were added. Membranes were incubated with Westar Supernova (Cyanogen, Bologna, Italy), and the luminescence was visualized and digitalized with C-DiGit Blot Scanner (Li-Cor Biosciences, Lincoln, NE, USA), and quantified with Image Studio Lite Software (v.5.2; Li-Cor). Phospho-proteins content was normalized to the corresponding total protein.

### Real-Time RT-PCR

Total RNA was extracted from the heart (LV), tibialis and soleus muscles with TRIzol Reagent. Reverse transcription was performed by using M-MLV Reverse transcriptase. cDNA was amplified with SensiFAST SYBR Hi-ROX PCR Master Mix using specific primers for Myh7, Ppargc1a, Opa1, Mfn2, Drp1, Mff, Ttn, Col1a1, Pabpn1, Titin, Ywhaz, HMBS, and GAPDH (Table 1) The Pfaffl comparative Ct method was used to analyze data (8). As described by Vesentini et al. to heart, values for each gene were normalized to Pabpn1 expression levels (9). HBMS, 18S, YWAS, GAPDH, and Pabpn1 genes, all candidate reference genes to skeletal muscle were validated to compare heart and skeletal muscle expression (Supplementary Figure 3).

**Table 1:** Primer sequence for RT-qPCR.

**Rat mRNAs**	**Forward**	**Reverse**
Titin	GCTGGTGAAGTCCAACTGACA	GGCTTAGTGAACTCAACCGGA
Opa1	CCGAAAGCCTCAGCTTGTTG	GCAGAAGTTCTTCCTGAAGTTGG
Mfn2	GTGACGTGTTGGGTGTGAT	GGACATCTCGTTTCTAGCTGGT
Ppargc1a	TGTGCAGCCAAGACTCTGTA	ACACCACTTCAATCCACCCA
Drp1	CGAAAACTGTCTGCCCGAGA	GCATTACTGCCTTTGGGACG
Mff	AGGTGACTCAATCTGGCACA	GCCCCACTCACCAAATGAGA
Myh7	GAGGAGAGGGCGGACATT	ACTCTTCATTCAGGCCCTTG
Col1a1	GGGATGGAGGGAGTTTACACG	CTTCATGTCCCGAGAGACGG
Ywhaz	ACTTGACATTGTGGACATCGGA	GTGGGACAGCATGGATGACA
HMBS	TCTAGATGGCTCAGATAGCATGCA	TGGACCATCTTCCTTGCTGAACA
GAPDH	CTACCCACGGCAAGTTCAAC	CCAGTAGACTCCACGACATAC
Pabpn	TATGGTGCGACAGCAGAAGA	TATGCAAACCCTTTGGGATG

### Statistical Analysis

Data are presented as mean ± standard deviation. Given the small sample size and the lack of normality of the data, we used Kruskal-Wallis with *post-hoc* Dunn's test to compare the effects of exercise in SHR and WYKR. A *p* < 0.05, two-tailed, was considered as significant. All analyses were performed using GraphPrism 8 (GraphPad, La Jolla, CA, USA).

## Results

### Blood Pressure and Heart Rate Measurements

**Figure 1:**
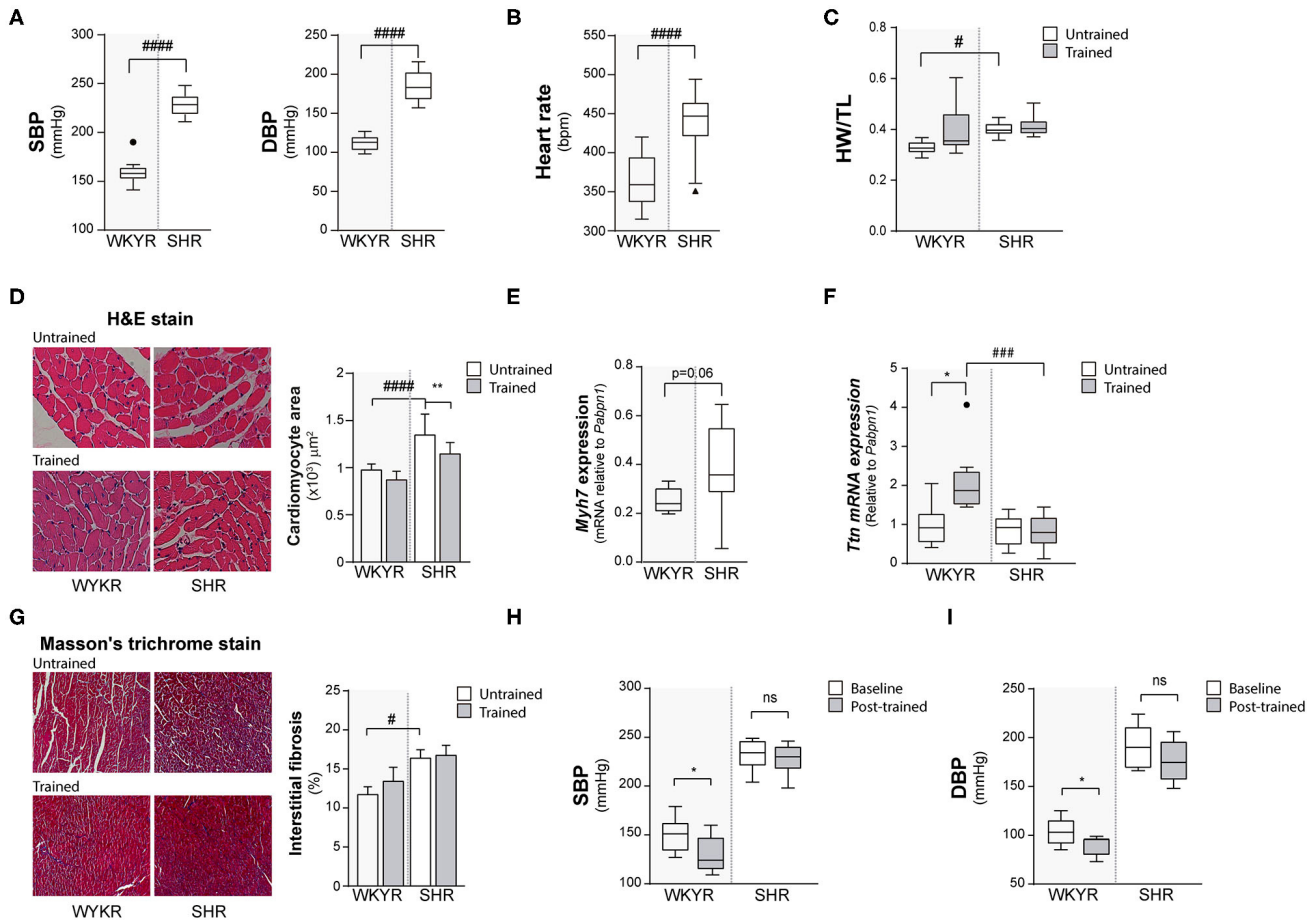
Hypertensive rat model validation. **(A)** Systolic and diastolic blood pressure (SBP and DBP) and **(B)** Heart rate values in WKYR and SHR 8 months old. **(C)** Heart weight/tibia length ratio as heart size parameter at protocol ending. **(D)** H&E staining, cardiomyocyte area and **(E)** myosin heavy chain 7 (Myh7) and **(F)** Titin (Ttn) expression as hypertrophy parameters in WKYR compared with SHR. **(G)** Masson's trichrome staining to determine interstitial fibrosis in SHR compared with WKYR after training. **(H,I)** Systolic and diastolic blood pressure at beginning (Baseline) and ending (Post-training) in WKYR and SHR. Groups: WYKR untrained (*n* = 8), WYKR trained (*n* = 8), SHR untrained (*n* = 10) and SHR trained (*n* = 10). All values are mean ± SD. Statistical significance was calculated using ANOVA, and group comparisons were performed using Tukey's test. *p < 0.05 untrained vs. trained group, **p < 0.01 untrained vs. trained group, ^#^*p* < 0.05, ^*###*^*p* < 0.001, and ^*####*^*p* < 0.0001 WKYR vs. SHR. Circles and triangles correspond to outlier data in WKYR and SHR, respectively.

Both SBP and DBP were higher in SHR than WKYR at baseline and after completing the study protocol (Figure 1A), irrespective of the allocated branch (trained or untrained) (SBP: WYKR 155 ± 15.67 vs. SHR 230 ± 14.04 mmHg, *p* < 0.0001; DBP: WYKR 107.8 ± 12.42 vs. SHR 188 ± 21.06 mmHg, *p* < 0.0001). Heart rate remained higher in SHR (367.9 ± 31.23 bpm vs. SHR 451.3 ± 27.95; *p* < 0.0001) throughout the study protocol (Figure 1B). Both SBP and DBP in the trained WYKR decreased respect to pre-training baseline. Conversely, in trained SHR, there was a non-significant decrease in DBP without changes in SBP (Figures 1H,I and Supplementary Figure 1C).

### Organ Weight

Heart size (assessed as heart weight/tibia length ratio) was significantly larger in SHR than in WKYR irrespective of the allocated branch (trained or untrained) (WYKR 0.327 ± 0.027 g/mm vs. SHR 0.400 ± 0.028 g/mm) (Figure 1C and Supplementary Figure 1A). Lung/body weight ratio was higher in both trained and untrained SHR when compared to WYKR, suggesting mild pulmonary congestion (Supplementary Figure 1B). The size of other organs did not differ between SHR and WKYR (data not shown).

### Histological Analysis

The increased heart size observed in SHR translated at the histological level into larger cardiomyocytes. Interestingly, trained SHR showed smaller cardiomyocytes when compared with untrained SHR (Figure 1D). The hypertrophic phenotype observed in SHR correlated with a higher expression of Myh7—a gene encoding the beta-myosin heavy chain— compared to WKYR (Figure 1E and Supplementary Figure 2A). The expression of other sarcomeric proteins, such as titin— related to cardiomyocyte passive stiffness (10), and modulation of active skeletal and cardiac contraction (11)— did not differ in untrained animals (Figure 1F), but was largely overexpressed in trained WKYR. Conversely, trained SHR did not exhibit differences in titin expression when compared with untrained animals (Figure 1F).

Fibrosis, a hallmark of pathological cardiac remodeling, was assessed using Masson's trichrome staining. As expected, basal collagen accumulation was higher in SHR when compared with WYKR, and there were no differences in both strains trained or untrained (Figure 1G). Tissue expression of the collagen-1 alpha-1 gene (Col1a1) followed the same pattern, with higher expression in SHR group (Supplementary Figure 2B).

### Echocardiography

**Figure 2:**
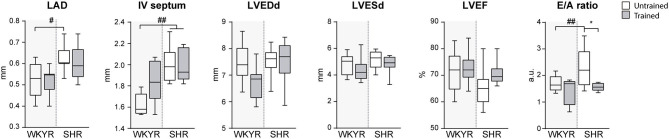
Ecochardiographic evaluation after completing the 8-week training period. LAD, Left atrial diameter; IV septum, Interventricular septum, LVEDd, Left ventricular end-diastolic diameter; LVESd, Left ventricular end systolic diameter; LVEF, Left ventricular ejection fraction; E/A ratio, Trans-mitral E wave/A wave ratio. Groups: WYKR untrained (*n* = 8), WYKR trained (*n* = 8), SHR untrained (*n* = 10) and SHR trained (*n* = 10). All values are mean ± SD. Statistical significance was calculated using ANOVA, and group comparisons were performed using Tukey's test. *p < 0.05 untrained vs. trained group, ^#^*p* < 0.05 WKYR vs. SHR and ^*##*^*p* < 0.01 WKYR vs. SHR.

The results of the echocardiographic evaluation performed after completing the 8-weeks training protocol are shown in Figure 2. As expected, SHR exhibited left atrial dilation and thicker interventricular septum suggesting early LV hypertrophy without significant enlargement when compared with normotensive WYKR. Systolic function, expressed as LVEF, was similar in WYKR and SHR irrespective of the training protocol. Interestingly, untrained SHR exhibited a significantly higher E/A ratio, a marker of diastolic dysfunction. Exercise normalized trans-mitral filling values (E/A ratio 2.43 ± 0.1 untrained SHR vs. 1.89 ± 0.16 trained SHR, *p* < 0.01) to those observed in WYKR controls.

### Mitochondrial Response to Exercise in Heart Tissue

**Figure 3:**
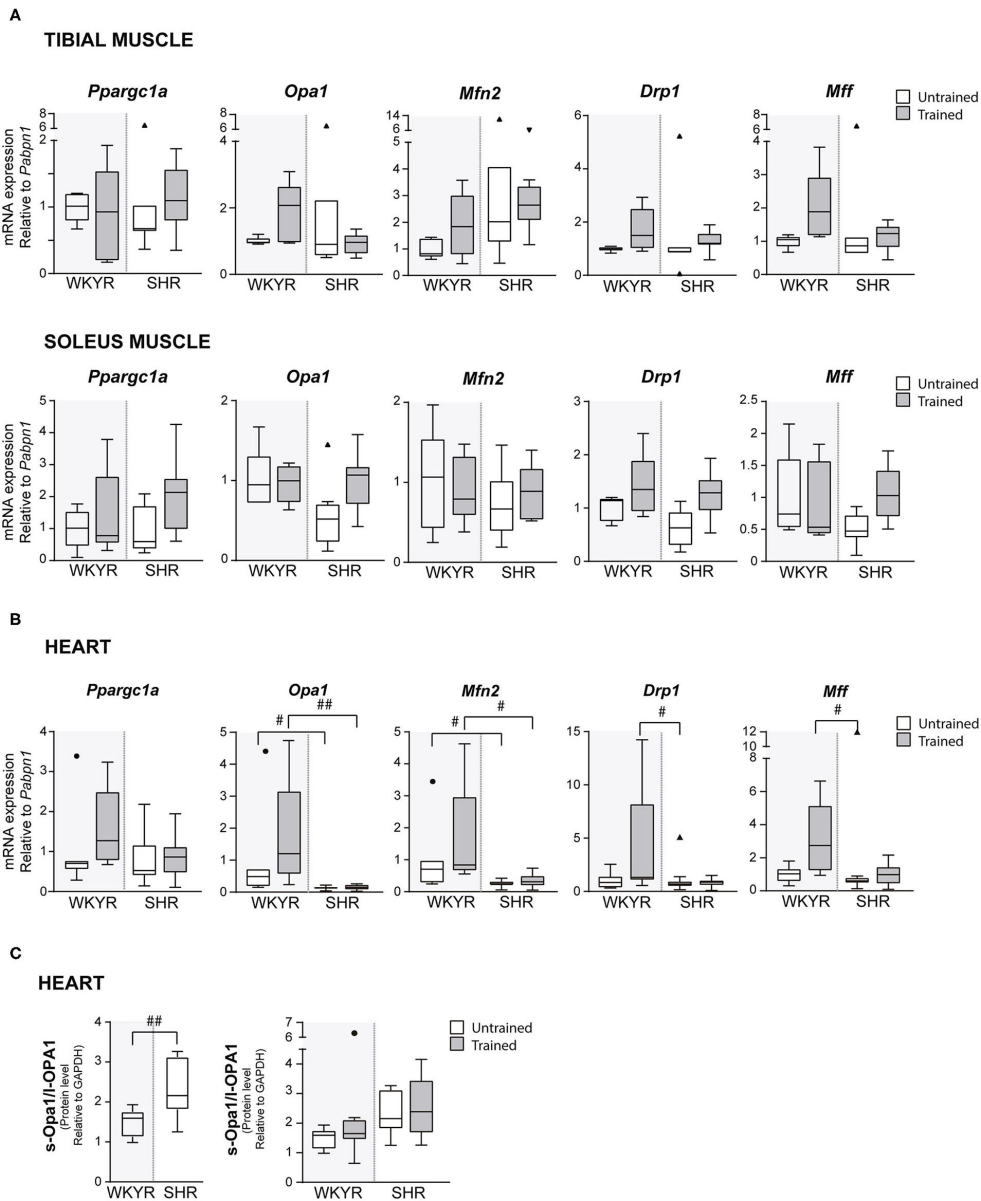
Mitochondrial modulators expression in WKYR and SHR. **(A)** Ppargc1a, Opa1, Mfn2, Drp1, and Mff mRNA levels were determined by RT-qPCR in tibial and soleus muscles total RNA extract at ending of exercise protocol. Values were normalized to Pabpn1 mRNA expression. **(B)** Ppargc1a, Opa1, Mfn2, Drp1, and Mff mRNA levels were determined by RT-qPCR in heart total RNA extract at ending of exercise protocol. Values were normalized to Pabpn1 mRNA expression. **(C)** s-OPA1/l-OPA1 protein ratio was determined by Western blot in total protein extracts of WKYR and SHR hearts and normalized by GAPDH levels. Groups: WYKR untrained (*n* = 8), WYKR trained (*n* = 6), SHR untrained (*n* = 10), and SHR trained (*n* = 10). All values of each group are presented as mean ± SD. Statistical significance was calculated using ANOVA, and group comparisons were performed using Tukey's test, ^#^*p* < 0.05 and ^*##*^*p* < 0.01 WKYR vs. SHR. Circles and triangles correspond to outlier data in WKYR and SHR, respectively.

To corroborate the metabolic effect of exercise, we determined the expression of Ppargc1a—the gene encoding PGC-1α, the master regulatory protein of mitochondrial biogenesis (12)—in tibialis and soleus muscles, where Ppargc1a overexpression has been reported as an exercise response marker (13). Surprisingly, we failed to see a significant increase in *Ppargc1a* expression in response to training in both strains, WKYR and SHR (*p* > 0.9, *p* = 0.14 (Figure 3A), but the chronic nature of the exercise—opposed to acute bouts of exercise—can explain the fail to induce Ppargc1a expression (14) (Supplementary Figure 4).

In order to determine if moderate exercise regulates cardiac metabolism in our model, we evaluated markers of mitochondrial physiology in the heart. First, we determined the expression of the Ppargc1a gene, which showed a tendency to increase in response to training in WKYR when compared with untrained SHR (*p* = 0.05) without significant differences between two trained groups (*p* = 0.30) (Figure 3B). We then evaluated the expression of genes related to mitochondrial dynamics, including genes involved in mitochondrial fusion (Opa1 and Mfn2) and fission (Drp1 and Mff) (15). We observed that Mfn2 and Opa1 levels were significantly higher in WHYR-UT compared to SHR-UT (*p* = 0.03 and *p* = 0.04, respectively), and this difference persisted after training (Mfn2 and Opa1 WKYR-T vs. SHR-T *p* = 0.01 and *p* = 0.002, respectively). The response of SHR to training was blunted (SHR-UT vs. SHR-T *p* > 0.9999 for all of the genes) (Table 2). These findings suggest that WYKR had a higher baseline expression of mitochondrial genes than SHR, which persists and becomes more evident in response to training. This impaired mitochondrial response may have critical consequences on metabolism and cardiovascular function.

**Table 2:** Statistics of gene expression in heart tissue of WKYR and SHR untrained and trained, *p*-value.

			**Dunn's multiple comparisons test**				
		***p*-value**	***p*-value**	***p*-value**	***p*-value**	***p*-value**	***p*-value**
**Gene**	**Kruskal-Wallis test**	**WKYR-UT vs. WKYR-T**	**SHR-UT vs. SHR-T**	**WKYR-UT vs. SHR-UT**	**WKYR-UT vs. SHR-T**	**WKYR-T vs. SHR-UT**	**WKYR-T vs. SHR-T**
drp1	0.0413	0.3112	>0.9999	>0.9999	>0.9999	0.0496	0.1601
mff	0.0077	0.3037	>0.9999	>0.9999	>0.9999	0.0047	0.0979
mfn2	0.0002	>0.9999	>0.9999	0.0315	0.3396	0.0003	0.0116
opa1	<0.0001	>0.9999	>0.9999	0.0401	0.1395	0.0003	0.002
ppargc	0.0497	0.2781	>0.9999	> 0.9999	>0.9999	0.0501	0.3099

*UT, Untrained; T, Trained*.

To confirm our findings, we evaluated the processing of OPA1, a protein that plays a critical role in the fusion of the inner mitochondrial membrane. In response to mitochondrial stress or impaired mitochondrial potential, OPA1 is cleaved, resulting in an increased abundance of short (soluble) forms of OPA1 and decreased abundance of in long (membrane-bound) forms. The increased s-OPA1/l-OPA1 ratio restricts fusion and can facilitate mitochondrial fission (16). Our results showed that OPA1 processing is higher in SHR vs. WKYR: SHR exhibited a significatively larger s-OPA1/l-OPA1 ratio, in trained individuals and close to being statistically significant (*p* = 0.05) in untrained individuals (Figure 3C).

### Transduction Pathways

**Figure 4:**
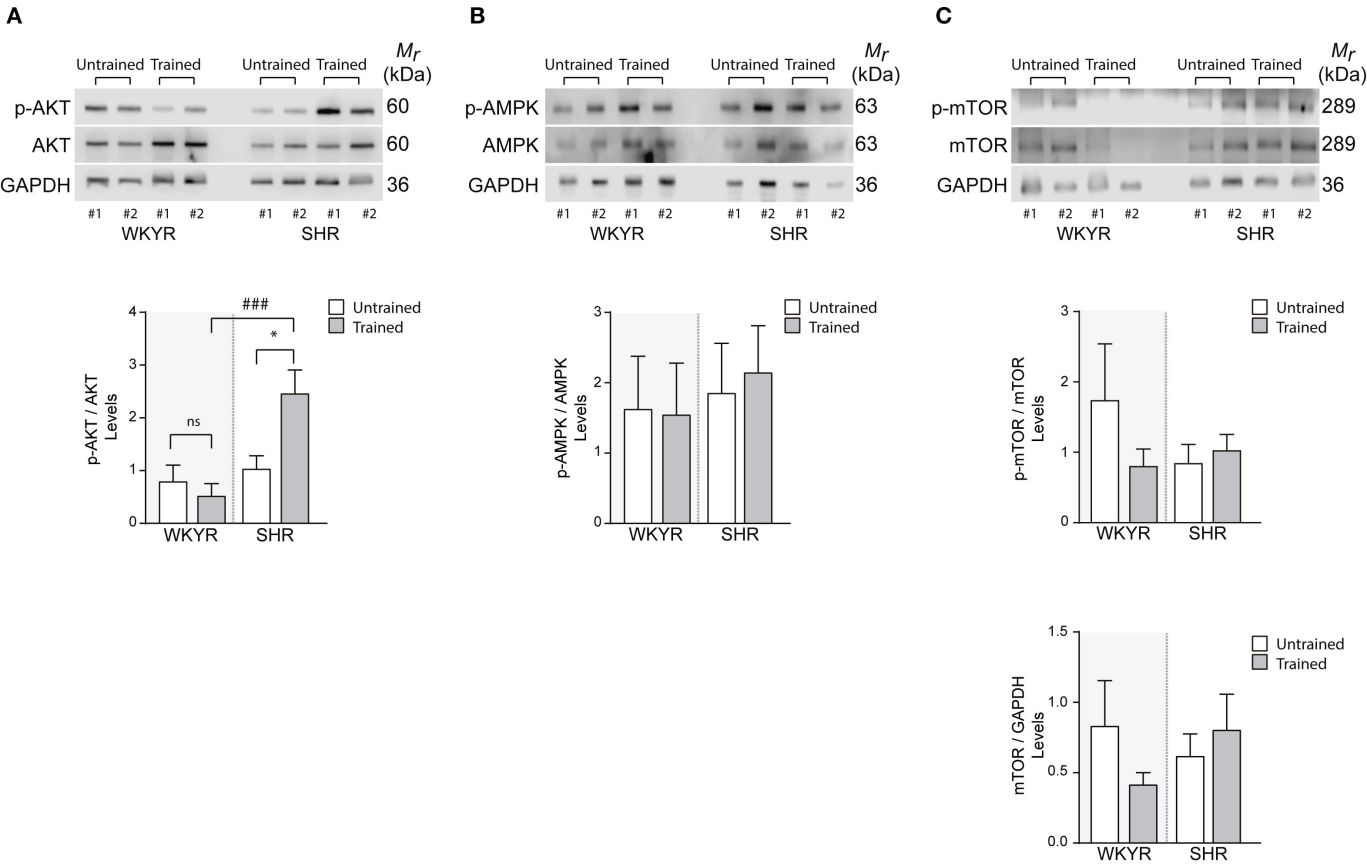
Metabolic and mitochondrial modulators activation in WKYR and SHR product of training program **(A)** phospho-AKT, **(B)** phospho-AMPK, and **(C)** phospho-mTOR was determined through Western blot in total protein extracts of WKYR and SHR hearts as an activation parameter and normalized by total AKT, AMPK, and mTOR, respectively. Additionally, in **(C)** we determined the total protein level of mTOR normalized by GAPDH levels. Images show two representative samples of each group and graphs include to the whole of samples for group. Groups: WYKR untrained (*n* = 8), WYKR trained (*n* = 8), SHR untrained (*n* = 10), and SHR trained (*n* = 10). All values are mean ± SD. Statistical significance was calculated using ANOVA, and group comparisons were performed using Tukey's test, *p < 0.05 untrained vs.f trained group, ^*###*^*p* < 0.001 WKYR vs. SHR. Circles and triangles correspond to outlier data in WKYR and SHR, respectively.

Since AMPK, AKT, and mTOR are modulators of cell metabolism and mitochondrial homeostasis (17–19), we wanted to determine whether their activation could explain the differences observed in the expression of mitochondrial genes in trained and untrained SHR-WKYR hearts. We assessed the phosphorylation of AKT, AMPK, and mTOR in total protein extracts from SHR and WKYR hearts through Western blot. Our results show that training increased the phosphorylation of AKT only in SHR, and had no effect in WKYR (p-AKT/AKT ratio 0.82 ± 0.87 vs. 0.54 ± 0.63 in WKYR untrained vs. trained, respectively; 1.05 ± 0.72 in SHR untrained vs. 2.48 ± 1.34 in SHR trained, *p* < 0.05) (Figure 4A).

Surprisingly, our training protocol did not increase the phosphorylation of AMPK in the heart of any of the studied groups (Figure 4B), a finding that can depend on the time elapsed from the last bout of exercise prior to euthanasia. Consistently, we observed that levels of phospho- and total-mTOR showed a small albeit non-significant decrease only in trained WKYR. Conversely, in SHR, the exercise protocol did not elicit any change in mTOR activation (Figure 4C).

## Discussion

The effect of exercise in blood pressure (BP) in SHR is well-characterized. Consistent with prior results, exercise training in our 8-months old animals was associated with a non-significant decrease of both SBP and DBP without reaching its normal values. While exercise has proved to significantly decrease BP in young animals (20), the effect is less evident in older animals and depends on the intensity, duration, and particularly of the age of start of the exercise training. While a reduction in BP is consistently reported in rats that start the exercise in the pre-hypertensive or early-hypertensive stage, but not in older animals (21).

As expected in a chronic hypertensive model, SHR had larger hearts, with increased wall thickness and larger systolic and diastolic diameters when compared with their normotensive counterparts. Exercise training did not reverse left ventricular hypertrophy or collagen volume fraction, a finding consistent with previously published evidence that exercise does not modify LV hypertrophy, and may even result in greater fibrosis and adverse remodeling depending on the training protocol (22). Surprisingly, we observed a significant reduction in cardiomyocyte hypertrophy, evidenced as a decreased myocyte transverse area. There are conflicting results in published papers where exercise training was unable to reverse cardiomyocyte hypertrophy in aged animals (23) while reducing the expression of pro-hypertrophic genes in young, pre-hypertensive rats (24). While these diverging results could be attributed to different animal age or exercise protocols, our findings should be interpreted with caution since we were unable to demonstrate any significant change in the expression of pro-hypertrophic genes such as Myh7.

One particularly interesting result is linked to titin expression. Titin is a giant scaffold protein that plays a critical role in sarcomere assembly. Normal response to exercise training implies a titin-related response that supports sarcomerogenesis; repeated exercise results in adaptive muscle remodeling and increased expression of structural sarcomeric proteins, including titin (25). While abnormal titin isoform ratio has already been described in SHR, resulting in higher passive tension upon stretch at a given sarcomere length and impaired cardiac performance (26), we are the first to report a blunted titin response to exercise in these animals.

Exercise in WYKR did not change any echocardiographic parameters. As expected, SHR presented dilated left atrium and left ventricular hypertrophy without over systolic dysfunction. This finding is consistent with previously published data that shows that SHR usually present normal or even enhanced myocardial performance up to 18 months of age, when the LV systolic function begins to decline, leading to ultimately to overt heart failure as a consequence of chronically increased afterload (27).

In our study, SHR exhibited a markedly increased E/A ratio, a marker of increased ventricular stiffness, elevated left ventricular filling pressure, and restrictive diastolic filling. Exercise induced a significant decrease in the E/A ratio which reached values similar to those observed in WYKR. Changes in diastolic function have been previously described in trained SHR and may reflect the effect of exercise on increased expression of SERCA2a and increased Thr-17 Phospholamban phosphorylation (28), leading to faster reuptake of diastolic calcium, and decreased left ventricular stiffness (29).

At the mitochondrial level, exercise is associated with improved mitochondrial homeostasis (30), increased expression of PGC-1α (31, 32), and increased mitochondrial fusion (33). Conversely, sedentarism leads to increased mitochondrial fission (34) and impaired mitochondrial quality control, with accumulation of deleterious mtDNA mutations (35), electron transport chain abnormalities (36), oxidative damage of mitochondrial proteins, and ultimately decreased mitochondrial respiration (37). In our model, exercise-induced PGC-1α expression in WYKR, coupled with an increase of several proteins involved in mitochondrial quality control and dynamics. Conversely, SHR did not show any significant activation of mitochondrial biogenesis in response to exercise.

Knock-out models have proved that PGC-1α is critical for maintaining normal mitochondrial function in the heart; PGC-1α knock-outs exhibit age-dependent systolic dysfunction, which became more apparent in response to stressors such as increased afterload due to hypertension (38, 39).

The increased transcription and nuclear translocation of PGC-1α in response to exercise depend on multiple upstream factors that translate mechanical and energy-related cues, including INF-γ, CAMK, NO, β-receptor signaling, p38-MAPK, AMPK, and p53 (12, 40). The lack of response observed in trained SHR may be partially explained by increased Akt activation: Akt directly phosphorylates PGC-1 α and prevents the recruitment of PGC-1α to its promoter regions in hepatocytes (41). Also, FoxO transcription factors contribute to the transcriptional regulation of PGC-1α; inactivation of FoxO3 by PI3K/Akt promotes PGC-1α downregulation in endothelial cells (42), and direct Akt-dependent FoxO1 phosphorylation can inhibit PGC-1α expression in hepatocytes (43). Contrasting with the cardioprotective effects of short-term Akt activation, sustained Akt activation disrupts mitochondrial bioenergetics in the heart, reducing mitochondrial oxidative capacity by reducing PGC-1α signaling and reducing FOXO-mediated transcriptional activation of mitochondrion-targeted nuclear genes (44). Interestingly, excessive Akt activation may explain gender-related differences in response exercise: in a long swim training protocol, female rats developed more severe hypertrophy than their male counterparts, which was associated with increased p-Akt/Akt ratio and decreased mTOR signaling (45).

In our model, we failed to show a consistent response to PGC-1α. As discussed above, this can be explained by the chronic nature of exercise, which can modify the epigenetic modulation of PGC-1α (14). Although several papers have addressed the role of histone modifications in the development of CVD, the evidence regarding modifications of the PGC-1α promoter is rather scarce (46). Recently, histone methylation marks in the PGC-1α locus have been reported in a murine model of high-salt induced heart failure. In this model, increased H3K9me3—a marker of gene repression—in the PGC-1α promoter was associated with impaired mitochondrial respiration. Interestingly, the use of an H3K9me3 inhibitor prolonged survival and restored mitochondrial function (47).

One of the unique characteristics observed in SHR was the abnormal OPA1 processing. A higher s-OPA1/l-OPA1 ratio promotes mitochondrial fragmentation and may promote a metabolic shift in the myocardium from its normal substrate (fatty acids) toward glucose, impaired mitochondrial energetics, energy depletion, and heart failure (48). Taken together, our results evidence a marked impairment in the mitochondrial adaptation to exercise in the heart of the SHR.

In summary, aerobic exercise improves diastolic parameters in SHR but fails to activate the cardiomyocyte mitochondrial adaptive response observed in non-hypertensive controls. This finding may explain the discrepancies on the effect of exercise in clinical settings and evidence of the need to further refine our understanding of the molecular response to physical activity in HTN subjects. The understanding of the interconnected signaling pathways orchestrating the mitochondrial adaptations to exercise in the heart represent potential targets for future pharmacological development.

## Data Availability Statement

The raw data supporting the conclusions of this article will be made available by the authors, without undue reservation, to any qualified researcher.

## Ethics Statement

The animal study was reviewed and approved by Comite Etico Cientifico Para el Cuidado de Animales y Ambiente, Unidad de Etica y Seguridad de la Investigacion, Pontificia Universidad Catolica de Chile.

## Author Contributions

The manuscript was drafted and written by CQ, GM, AC, AT, and HV. GM and IO conducted the exercise training protocol. GM performed all RT-PCR experiments. IO, AT, DV-I, and MC extracted protein from tissue samples and performed the WB studies. LG contributed the echocardiography data and evaluation of the animals. PC, DV-I, and AC reviewed the final manuscript and contributed to the discussion. HV planned and evaluated the overall experimental design. All authors have contributed parts of it, as well as read, and edited the final submitted version.

## Conflict of Interest

The authors declare that the research was conducted in the absence of any commercial or financial relationships that could be construed as a potential conflict of interest.

## Supplementary Material

Supplementary Figure 1 Training effect on hypertensive rat model. **(A)** Heart weight/ Body weight ratio and **(B)** Lung weight/ Body ratio in WKYR and SHR after training program. **(C)** Systolic and diastolic blood pressure (SBP and DBP) and Heart rate values in WKYR and SHR divided by groups at beginning and final of training. Groups: WYKR untrained (*n* = 8), WYKR trained (*n* = 8), SHR untrained (*n* = 10), and SHR trained (*n* = 10). All values are mean ± SD. Statistical significance was calculated using ANOVA, and group comparisons were performed using Tukey's test. ^*##*^*p* < 0.01, ^###^*p* < 0.001, and ^####^*p* < 0.0001 WKYR vs. SHR. Circles correspond to outlier data in WKYR.

Supplementary Figure 2 Cardiac markers expression in WKYR and SHR. **(A)** Myosin heavy chain 7 (Myh7) and **(B)** Collagen 1-alpha1 (Col1a1) mRNA levels were determined by RT-qPCR in heart total RNA extract at ending of training program. Values were normalized to Pabpn1 mRNA expression. Groups: WYKR untrained (*n* = 8), WYKR trained (*n* = 8), SHR untrained (*n* = 10), and SHR trained (*n* = 10). All values are mean ± SD. Statistical significance was calculated using ANOVA, and group comparisons were performed using Tukey's test, #*p* < 0.05 WKYR vs. SHR. Circles and triangles correspond to outlier data in WKYR and SHR, respectively.

Supplementary Figure 3 Validation of housekeeping genes as reference for RT-qPCR gene expression in skeletal muscle. Plot indicating Ct values of HBMS, 18S, YWAS, GAPDH, and Pabpn1, all candidate reference genes to skeletal muscle. We choose to use Pabpn1 to compare heart and skeletal muscle expression.

Supplementary Figure 4 Mitochondrial modulators expression in WKYR and SHR. Ppargc1a, Opa1, Mfn2, Drp1, and Mff mRNA levels were determined by RT-qPCR in **(A)** tibial and **(B)** soleus muscles total RNA extract at ending of exercise protocol. Values were normalized to HBMS mRNA expression. Groups: WYKR untrained (*n* = 8), WYKR trained (*n* = 8), SHR untrained (*n* = 10), and SHR trained (*n* = 10). All values are mean ± SD. Statistical significance was calculated using ANOVA, and group comparisons were performed using Tukey's test. Triangles correspond to outlier data in SHR.
