# Supplementary figures and images for: Moderate Exercise in Spontaneously Hypertensive Rats Is Unable to Activate the Expression of Genes Linked to Mitochondrial Dynamics and Biogenesis in Cardiomyocytes
Source: Front Endocrinol (Lausanne). 2020 Aug 19;11:546. doi: 10.3389/fendo.2020.00546 (PMC7466645; doi:10.3389/fendo.2020.00546)

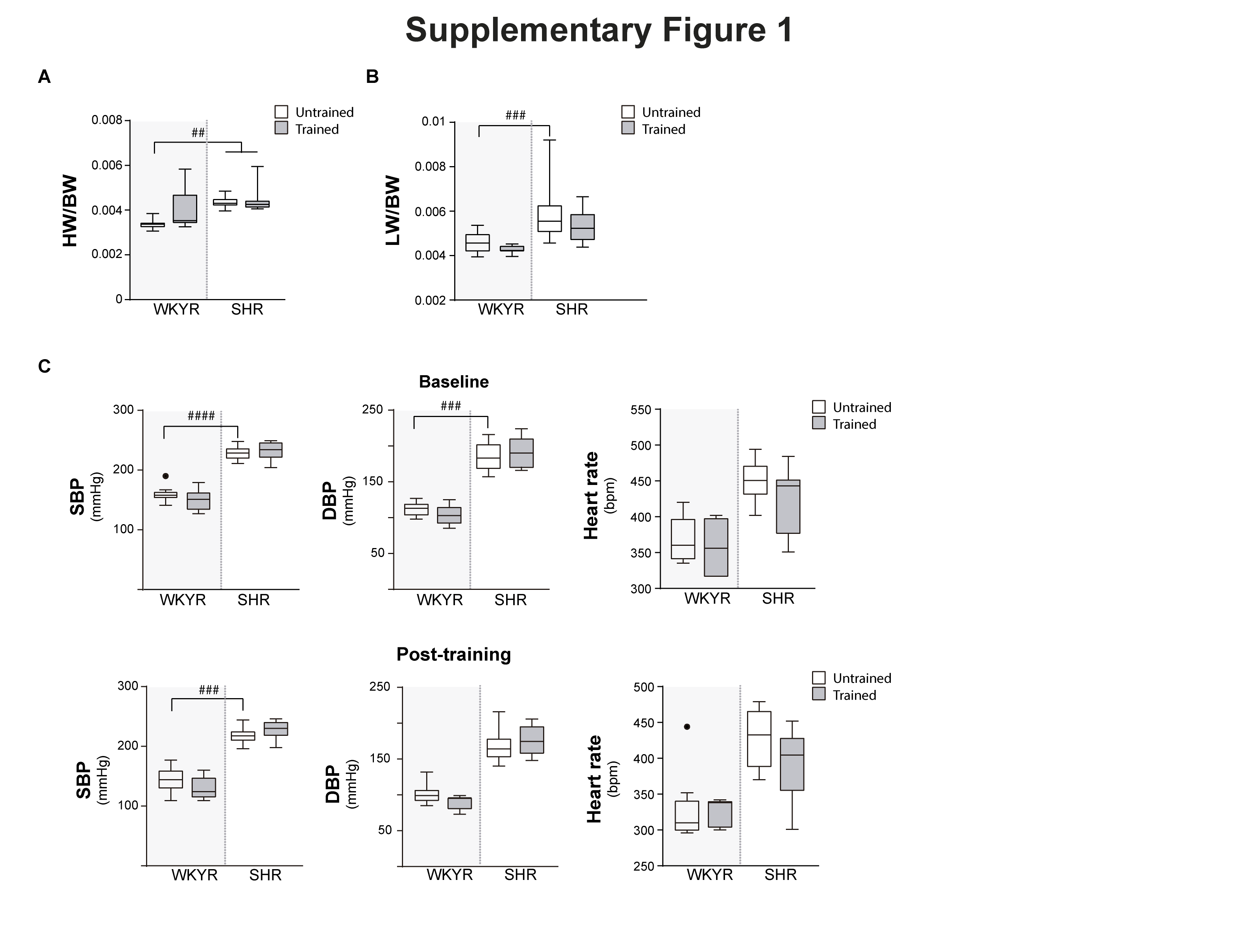

Supplement: Supplementary Figure 1 — Training effect on hypertensive rat model. (A) Heart weight/ Body weight ratio and (B) Lung weight/ Body ratio in WKYR and SHR after training program. (C) Systolic and diastolic blood pressure (SBP and DBP) and Heart rate values in WKYR and SHR divided by groups at beginning and final of training. Groups: WYKR untrained (n = 8), WYKR trained (n = 8), SHR untrained (n = 10), and SHR trained (n = 10). All values are mean ± SD. Statistical significance was calculated using ANOVA, and group comparisons were performed using Tukey's test. ##p < 0.01, ###p < 0.001, and ####p < 0.0001 WKYR vs. SHR. Circles correspond to outlier data in WKYR. [file Image_1.TIF]

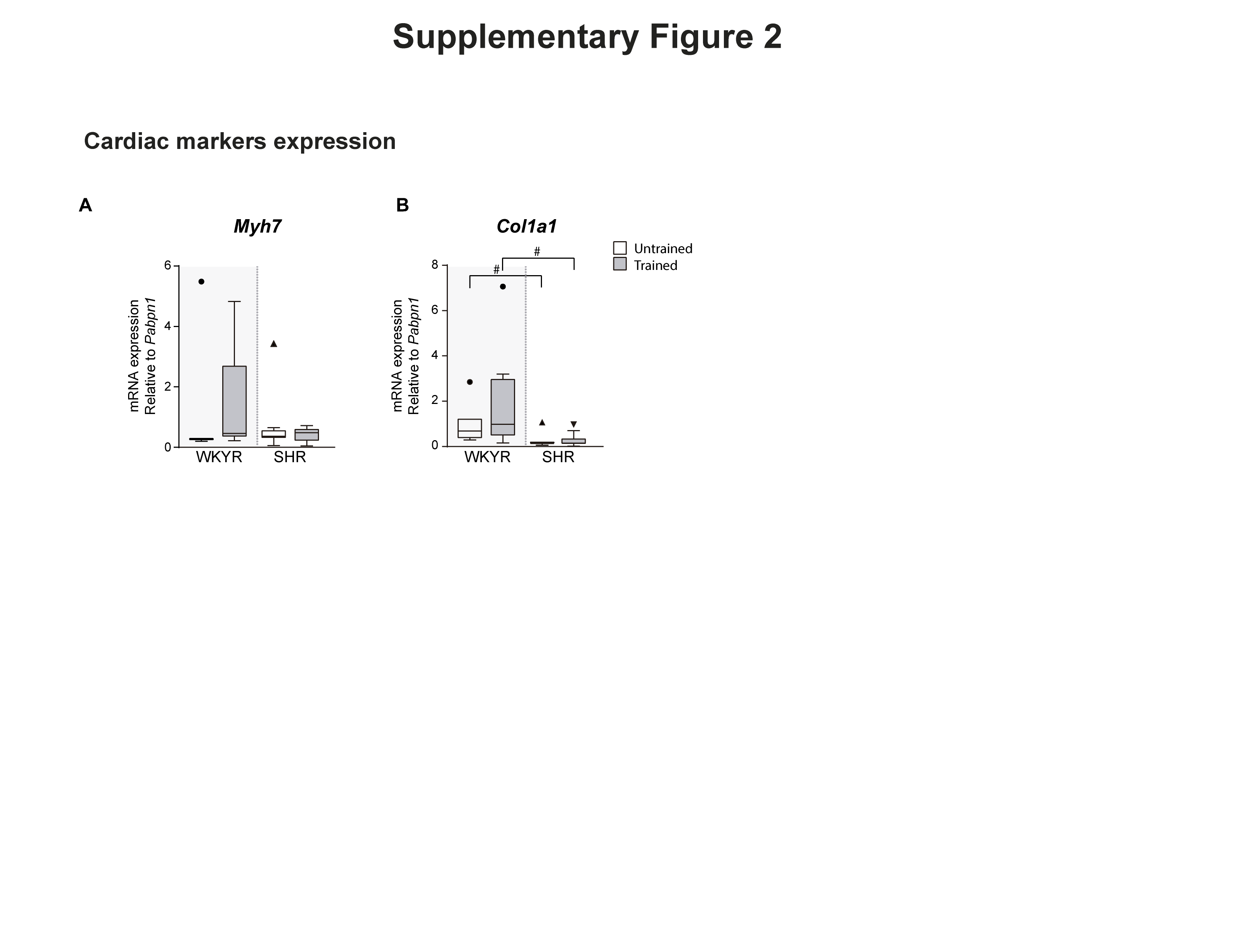

Supplement: Supplementary Figure 2 — Cardiac markers expression in WKYR and SHR. (A) Myosin heavy chain 7 (Myh7) and (B) Collagen 1-alpha1 (Col1a1) mRNA levels were determined by RT-qPCR in heart total RNA extract at ending of training program. Values were normalized to Pabpn1 mRNA expression. Groups: WYKR untrained (n = 8), WYKR trained (n = 8), SHR untrained (n = 10), and SHR trained (n = 10). All values are mean ± SD. Statistical significance was calculated using ANOVA, and group comparisons were performed using Tukey's test, #p < 0.05 WKYR vs. SHR. Circles and triangles correspond to outlier data in WKYR and SHR, respectively. [file Image_2.TIF]

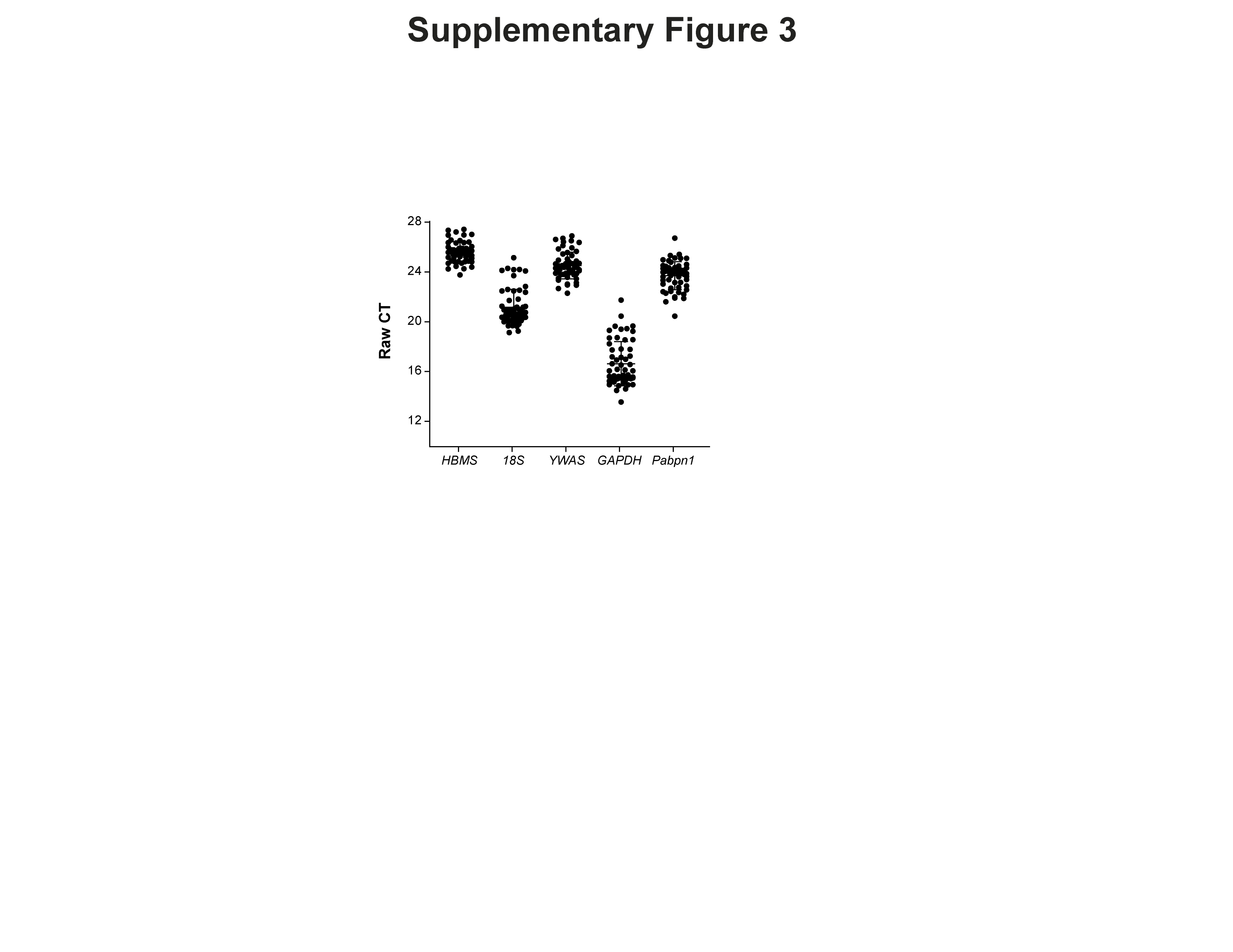

Supplement: Supplementary Figure 3 — Validation of housekeeping genes as reference for RT-qPCR gene expression in skeletal muscle. Plot indicating Ct values of HBMS, 18S, YWAS, GAPDH, and Pabpn1, all candidate reference genes to skeletal muscle. We choose to use Pabpn1 to compare heart and skeletal muscle expression. [file Image_3.TIF]

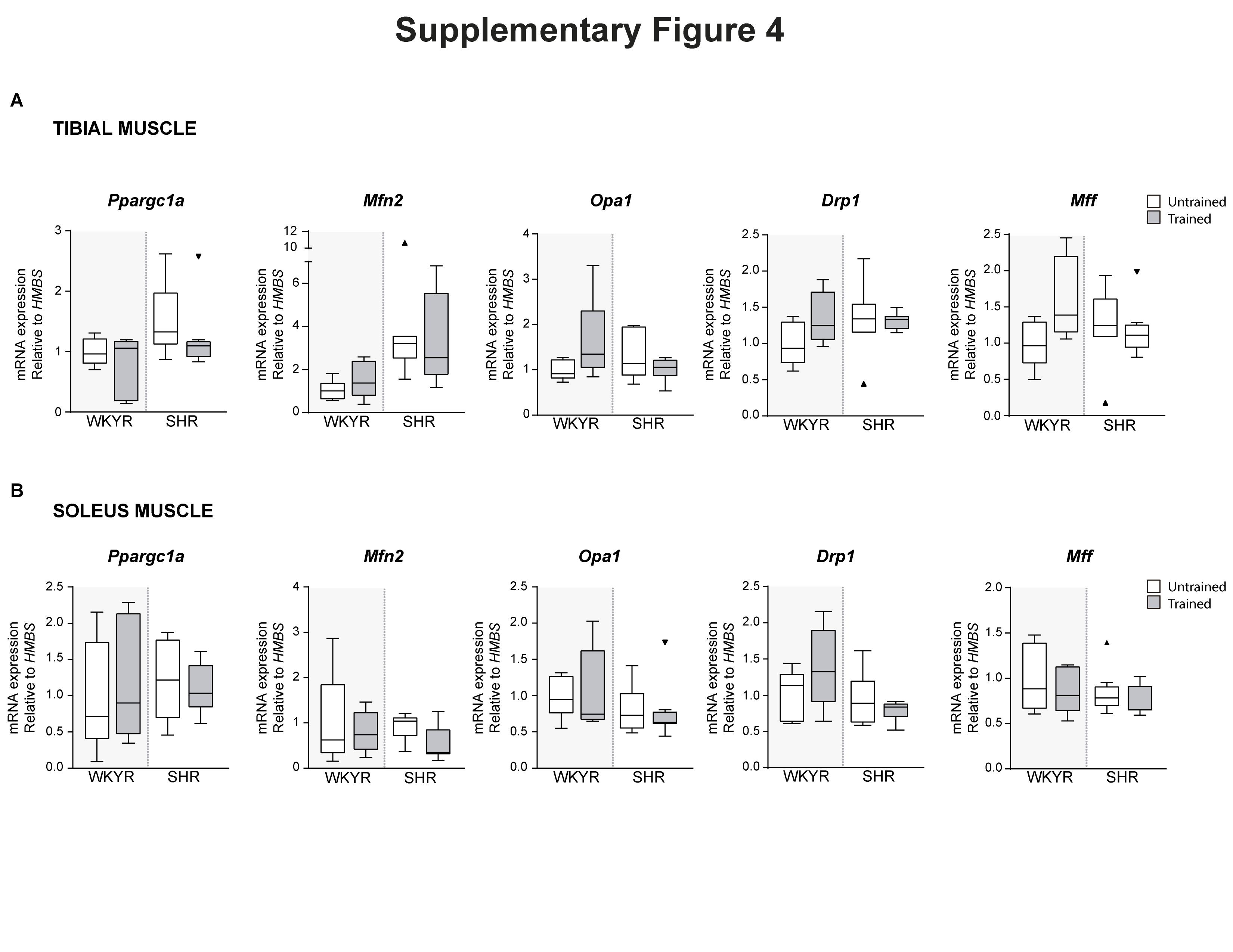

Supplement: Supplementary Figure 4 — Mitochondrial modulators expression in WKYR and SHR. Ppargc1a, Opa1, Mfn2, Drp1, and Mff mRNA levels were determined by RT-qPCR in (A) tibial and (B) soleus muscles total RNA extract at ending of exercise protocol. Values were normalized to HBMS mRNA expression. Groups: WYKR untrained (n = 8), WYKR trained (n = 8), SHR untrained (n = 10), and SHR trained (n = 10). All values are mean ± SD. Statistical significance was calculated using ANOVA, and group comparisons were performed using Tukey's test. Triangles correspond to outlier data in SHR. [file Image_4.TIF]
